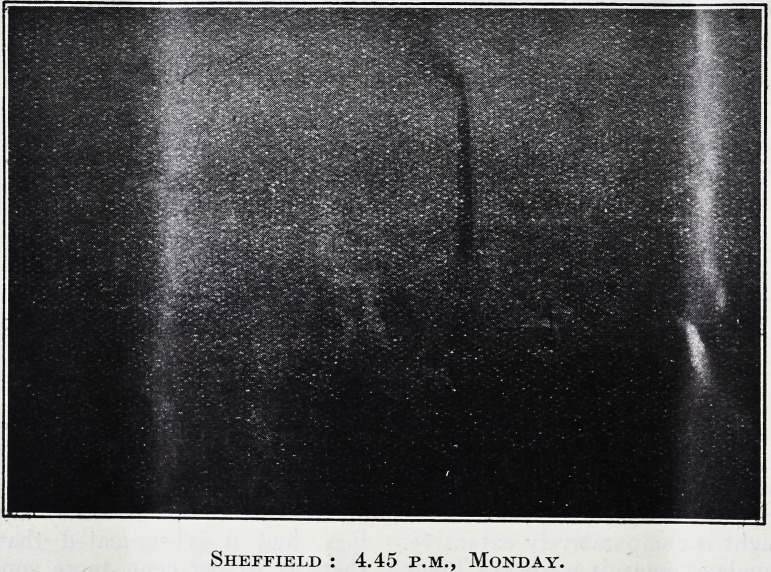# Sheffield White and Sheffield Black

**Published:** 1924-03

**Authors:** 


### SHEFFIELD WHITE AND SHEFFIELD BLACK.

### THE TRAGEDY OF MONDAY.

In Sheffield Sunday is a day of rest in more senses than one. Not only do men—or most of them—cease from their labours, but the factory chimneys refrain from belching forth their volumes of black smoke laden with innumerable tons of impurities destructive of things much more precious than buildings and vegetation. Health and life itself are imperilled by the impenetrable gloom which broods over the city from Monday morning to Saturday night, to be lightened only on Sunday. Our three illustrations, for which we are indebted to the courtesy of the British Commercial Gas Association, show that at church-time on a Sunday morning in mid-September, and no doubt at other seasons, Sheffield is no worse a town to live in than Birmingham or Manchester, Leeds or Newcastle. The landscape may not be alluring nor the architecture picturesque, but such things are not expected in such an environment (though some day they will be). The sky is clear and, with the observer perched high up, the line of sight is comparatively extensive.

Sheffield : 11 a.m., Sunday.

When, however, Monday comes, it is always Black Monday in Sheffield. By breakfast-time the horizon is already shadowed, though the sun is still making a fight for it. By lunch-time the ceaseless outpourings of hundreds of chimneys have created a thick smoky fog, and by tea-time Sheffield has to be taken on trust. The photographic illustrations represent the happenings of no special day, as they show us Sheffield as it is every day, year in and year out, with such ameliorations as wind and weather may sometimes provide. Unhappily this state of things is by no means a Sheffield "peculiar." It exists, with greater or less severity, in every great manufacturing town, and often in an almost continuous chain of towns. The effect upon the health and spirits of the population of this shutting out of the sun is necessarily exceedingly lowering and depressing, and it is amazing that the national commonsense upon which we pride ourselves has so long allowed its continuance. Other countries, and especially the United States, are less patient. Pittsburgh, for instance, the great centre of the steel industry, used to be described as "Hell with the lid off," and was probably one of the dirtiest places on earth. Its people set to work to remedy matters, and now Pittsburgh, with its enormous production of steel, is cleaner than Nottingham with its ace manufacture.

The number of deaths from pulmonary and cardiac diseases is shown to increase in direct proportion to an increase in the intensity and duration of smoke fogs, and it is estimated that, broadly speaking, there is 20 per cent. more sunlight in the country than in a smoky town and, of course, infinitely more than that in comparison with a city like Sheffield. Moreover, the damage caused by smoke to vegetation and agriculture is very severe, and in and near industrial centres dead and dying trees are a sad feature of the countryside. Yet all these evil things might be ended with comparative ease! If, instead of burning crude coal in this barbarous manner, the coal were distilled in the closed retorts of the gas-works,

Sheffield : Noon, Monday.

this unhealthy and dispiriting smoke pall would disappear. No only so, but the very substances which form its filthy body, instead of being agents of destruction, would be turned instead into sources of health and wealth and beauty. Out of smoke would come, as by a fairy's wand, dyes and drugs, fertilisers and perfumes. We have only to use the means at our disposal and we can make this modern fairy tale come true.

Sheffield : 4.45 p.m., Monday.

## Figures and Tables

**Figure f1:**
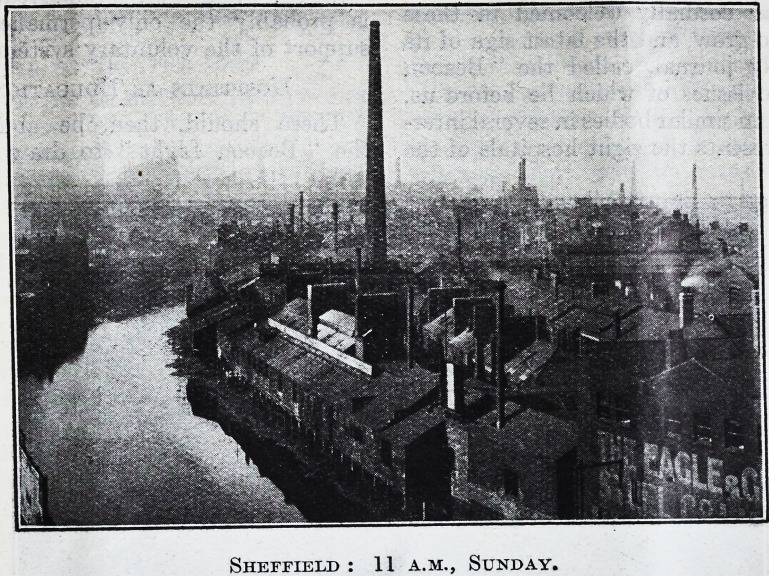


**Figure f2:**
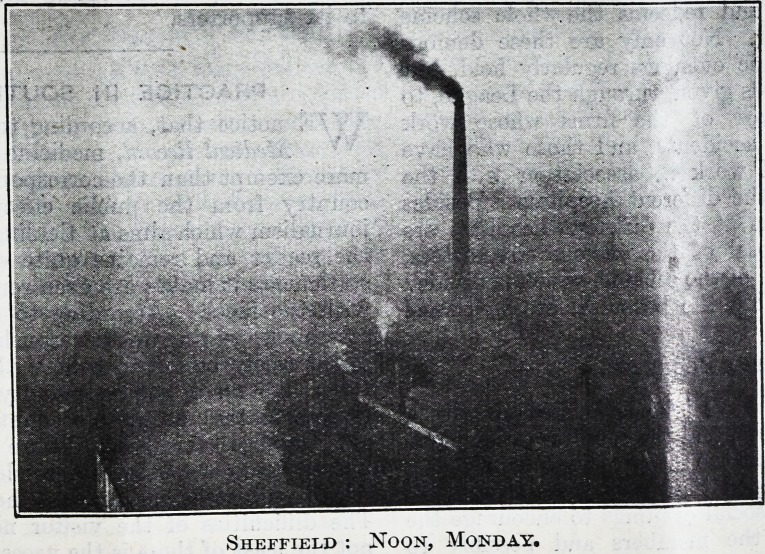


**Figure f3:**